# Type 2 diabetes in midlife and risk of cerebrovascular disease in late life: a prospective nested case−control study in a nationwide Swedish twin cohort

**DOI:** 10.1007/s00125-019-4892-3

**Published:** 2019-06-05

**Authors:** Rongrong Yang, Nancy L. Pedersen, Cuiping Bao, Weige Xu, Hui Xu, Ruixue Song, Xiuying Qi, Weili Xu

**Affiliations:** 1 Department of Epidemiology and Biostatistics, School of Public Health, Tianjin Medical University, Qixiangtai Road 22, Heping District, 300070 Tianjin, People’s Republic of China; 2 Department of Medical Epidemiology and Biostatistics, Karolinska Institutet, Stockholm, Sweden; 3 Department of Psychology, University of Southern California, Los Angeles, CA USA; 4 Department of Radiology, Tianjin Union Medical Centre, Tianjin, People’s Republic of China; 5 Department of Radiology, Tianjin Gongan Hospital, Tianjin, People’s Republic of China; 6 Aging Research Center, Department of Neurobiology, Care Sciences and Society, Karolinska Institutet, Tomtebodavägen 18A Floor 10, SE-171 65 Solna, Stockholm Sweden

**Keywords:** Cerebrovascular disease, Prospective nested case−control study, The Swedish twin cohort, Type 2 diabetes

## Abstract

**Aims/hypothesis:**

We aimed to examine the association between midlife type 2 diabetes mellitus and cerebrovascular disease (CBD) in late life, and further to explore whether genetic and early-life familial environmental factors (such as shared childhood socioeconomic status and adolescent environment) play a role in this association.

**Methods:**

In this prospective nested case−control study based on the Swedish Twin Registry, 33,086 twin individuals who were born in 1958 or earlier and were CBD-free before the age of 60 were included. Midlife (40–59 years) type 2 diabetes was ascertained from self-report, the National Patient Registry (NPR) and glucose-lowering medication use. CBD diagnosis (cerebral infarction, occlusion of cerebral arteries, subarachnoid haemorrhage, intracerebral haemorrhage and unspecified CBD) and onset age were identified from the NPR. Late-life CBD was defined as CBD onset age ≥60 years. Generalised estimating equation (GEE) models were used to analyse unmatched case−control data (adjusted for the clustering of twins within a pair). Conditional logistic regression was used in co-twin matched case−control analyses in CBD-discordant twin pairs.

**Results:**

Of all the participants, 1248 (3.8%) had midlife type 2 diabetes and 3121 (9.4%) had CBD in late life. In GEE models adjusted for age, sex, education, BMI, smoking, alcohol consumption, marital status, hypertension and heart disease, the ORs (95% CIs) of type 2 diabetes were 1.29 (1.03, 1.61) for cerebral infarction, 2.03 (1.20, 3.44) for occlusion of cerebral arteries, 0.52 (0.12, 2.21) for subarachnoid haemorrhage and 0.78 (0.45, 1.36) for intracerebral haemorrhage. In multi-adjusted conditional logistic regression, the OR of the type 2 diabetes–cerebral infarction association was 0.96 (0.51, 1.80). The differences in ORs from the GEE and co-twin control analyses were not statistically significant (*p* = 0.780).

**Conclusions/interpretation:**

Midlife type 2 diabetes is significantly associated with increased risk of cerebral infarction and occlusion of cerebral arteries, but not intracerebral haemorrhage or subarachnoid haemorrhage in late life. Genetic and early-life familial environmental factors do not appear to account for the type 2 diabetes–cerebral infarction association, but further clarification is needed.

**Electronic supplementary material:**

The online version of this article (10.1007/s00125-019-4892-3) contains peer-reviewed but unedited supplementary material, which is available to authorised users.

## Introduction

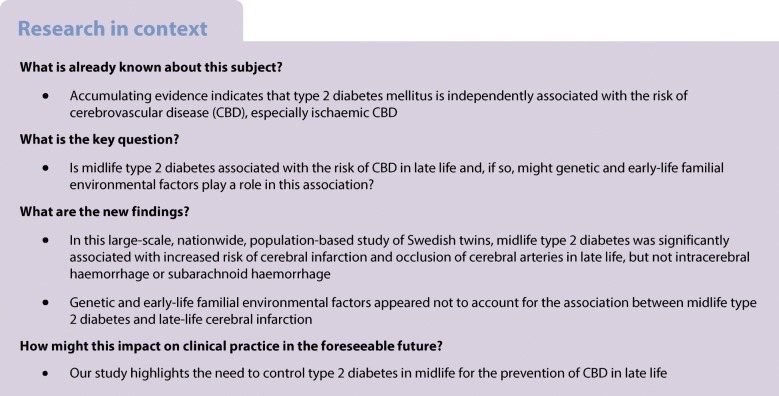



Worldwide, cerebrovascular disease (CBD) and type 2 diabetes mellitus are common disorders that are among the top ten leading causes of death, killing approximately eight million people in 2016 [1]. CBD includes a variety of medical conditions that affect the blood vessels of the brain and cerebral circulation. There are two main classifications of CBD: ischaemic and haemorrhagic. Thus far, accumulating evidence suggests that type 2 diabetes is independently associated with the risk of CBD, especially ischaemic CBD [2–8]. In a collaborative meta-analysis of 102 prospective studies including 698,782 participants, the adjusted HR was 2.27 (CI 1.95, 2.65) for ischaemic stroke in people with vs those without type 2 diabetes [9]. Type 2 diabetes may cause atherosclerotic changes in the cerebral arteries, and lead to ischaemic stroke [10]. Several studies have examined the association between type 2 diabetes and haemorrhagic CBD, but with inconsistent results [3, 5, 8, 9, 11–19]. Furthermore, only a few cohort studies have examined the relationship between type 2 diabetes and intracerebral haemorrhage and subarachnoid haemorrhage individually [12, 20]. To our knowledge, no studies have focused on the association between midlife type 2 diabetes and CBD in late life.

Both type 2 diabetes and CBD are complex genetic and lifestyle-related disorders. Genetic and familial environmental factors (such as fetal environment, maternal smoking or childhood socioeconomic status) are involved in the development of type 2 diabetes [21] and CBD [22, 23], but their contribution to the association between type 2 diabetes and CBD is unknown. In population-based studies that address the association between type 2 diabetes and CBD, some major confounders can be controlled for in data analysis, but not familial factors (such as genetic background and early-life environmental factors shared by family members). Twin studies provide an opportunity to minimise potential confounding due to unmeasured genetic predisposition and shared familial environment in childhood or adolescence. Twins are generally raised together and share the same genetic background as well as intrauterine, childhood and adolescent environments. As naturally matched pairs, the confounding effects of genetics and familial environments are removed when comparisons are made within the pair. Thus, co-twin case−control analyses can be more informative than unrelated case−control analyses [24, 25].

In the current study we aimed to: (1) examine the associations between midlife type 2 diabetes (i.e. type 2 diabetes with an onset between 40 and 59 years of age) and risk for CBD in late life; and (2) explore whether genetic and familial environmental factors could explain the type 2 diabetes–CBD association using data from a nationwide cohort of Swedish twins.

## Methods

### Study population

**Fig. 1:**
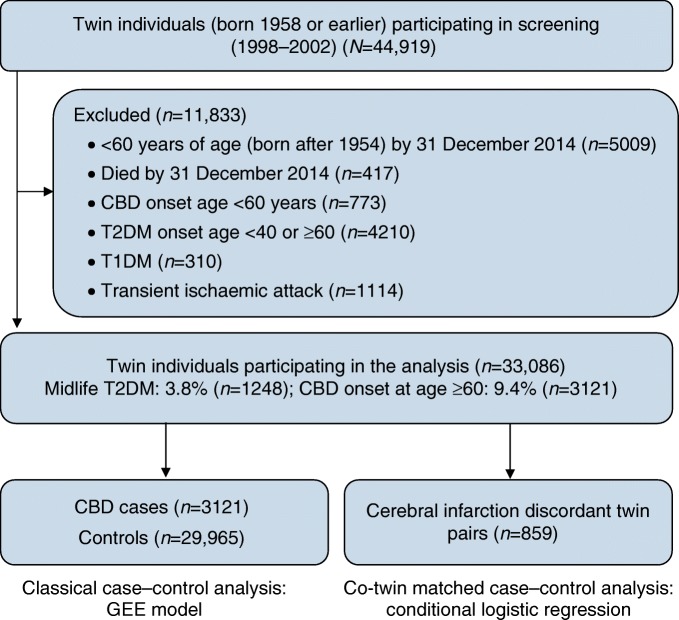
Flow chart of the study population. T1DM, type 1 diabetes mellitus; T2DM, type 2 diabetes mellitus

This prospective, nested case−control study included twins from the nationwide Swedish Twin Registry (STR), which was started in the 1960s [26]. In 1998–2002, all living twins (≥40 years of age, i.e. born in 1958 or earlier) in the registry were invited to participate in the Screening Across the Lifespan Twin study (SALT), a full-scale screening that gathered data on an extended set of variables via computer-assisted telephone interview. Out of 44,919 twin individuals eligible for the interview, 5009 who had not reached the age of 60 by the end of available follow-up on 31 December 2014 (born after 1954), 417 who died by 31 December 2014, 773 who had CBD before the age of 60 years, 4210 who had type 2 diabetes with onset age <40 years or ≥60 years, 310 who had type 1 diabetes, and 1114 who had transient ischaemic attack were excluded. Finally, 33,086 individuals remained for the current analyses (Fig. 1).

### Data collection

Information on demographics (age, sex and educational attainment), lifestyle (smoking, alcohol consumption), anthropometric measures (weight and height), zygosity and medication use (including treatment for type 2 diabetes) was obtained from the SALT survey [26]. Education was defined by the maximum years of formal schooling attained, and dichotomised into <8 vs ≥8 years. BMI was calculated as weight (kg) divided by height squared (m^2^), and categorised as <20 kg/m^2^ (underweight), 20–24.9 kg/m^2^ (normal weight), 25–29.9 kg/m^2^ (overweight), and ≥30 kg/m^2^ (obese). Smoking status was categorised as never smoked, former smoker and current smoker. Alcohol consumption was dichotomised as never consumed alcohol vs past/current consumer of alcohol. Marital status was defined as married/cohabitating vs single (including divorced).

Information on history of type 2 diabetes, CBD, hypertension and heart disease (including coronary heart disease, cardiac arrhythmias and heart failure) was derived from the National Patient Registry (NPR), which covers all inpatient care in Sweden since the 1960s up to the end of 2014. Each medical record in the NPR included up to eight discharge diagnoses recorded as International Classification of Disease (ICD) codes. The seventh revision (ICD-7) was used through to 1968, the eighth revision (ICD-8) from 1969 through to 1986, the ninth revision (ICD-9 [www.icd9data.com/2007/Volume1]) from 1987 through to 1996 and the tenth revision (ICD-10 [http://apps.who.int/classifications/icd10/browse/2016/en]) since 1997 through to the end of available follow-up in 2014.

Informed consent was required from all participants. The data collection procedures were approved by the Regional Ethics Committee at Karolinska Institutet, Stockholm, Sweden, and the Institutional Review Board of the University of Southern California, USA.

### Ascertainment of diabetes

Both type 1 and type 2 diabetes were ascertained based on self- and informant-reported history of type 2 diabetes, glucose-lowering medication use, or NPR (ICD-7 code 260, ICD-8 and -9 code 250, and ICD-10 codes E10–E14). The age at type 2 diabetes onset was estimated according to the earliest recorded date of type 2 diabetes in the NPR or the self-reported date of type 2 diabetes onset available in SALT. Midlife type 2 diabetes was defined as type 2 diabetes onset at age 40–59 years. Information on treatment of type 2 diabetes was collected from the SALT survey and the Swedish Drug Registry (The Anatomical Therapeutic Chemical code A10) since 2006. Treatment of type 2 diabetes was categorised as diet, oral glucose-lowering drugs, insulin and combined oral glucose-lowering drugs and insulin.

### Assessment of CBD

Information on CBD diagnosis (ICD-7 codes 330–334, ICD-8 codes 430–438, ICD-9 codes 430–437, ICD-10 codes I60–I68) was derived from NPR records between 1960 and 2014. The CBD subtypes in the current study included: (1) cerebral infarction; (2) occlusion and stenosis of precerebral or cerebral arteries not resulting in cerebral infarction (termed as occlusion of cerebral arteries below); (3) subarachnoid haemorrhage; (4) intracerebral haemorrhage; and (5) unspecified CBD [27]. The age of CBD onset was estimated according to the earliest recorded date of the CBD diagnosis in NPR.

### Statistical analyses

The characteristics of the study population by midlife type 2 diabetes or CBD were compared using χ^2^ tests for categorical variables, unpaired Student’s *t* test for continuous variables with normal distribution, and Mann–Whitney *U* test for continuous variables with non-normal distribution. Generalised estimating equation (GEE) models were used in unmatched case−control analyses, controlling for the clustering of twins within a pair. In unmatched case−control analysis, the basic-adjusted models were adjusted for age, sex and education. The multi-adjusted models were further adjusted for BMI, smoking, alcohol consumption, marital status, hypertension and heart disease. Data for the co-twin matched case−control study were analysed using conditional logistic regression, in which twin pairs who were discordant for outcome. In the co-twin control design, the disease-free co-twin (in both monozygotic and dizygotic twin pairs) is used as a control for the diseased twin. Using discordant twin pairs is more informative than using unrelated case−control samples, as cases and controls matched for genetic background and familial environmental factors such as fetal environment, maternal smoking or childhood socioeconomic status [28, 29]. In the co-twin control analysis, the basic-adjusted models were adjusted for sex and education. The multi-adjusted models were further adjusted for BMI, smoking, alcohol consumption, marital status, hypertension and heart disease.

Logistic regression was used to test the difference in ORs from the GEE model and conditional logistic regression by examining the difference in midlife type 2 diabetes between unmatched and co-twin controls [30]. If an OR for the observed association in the unmatched case−control analysis becomes strengthened or attenuated (or even disappears) in co-twin control analyses, and the difference in ORs from the GEE and conditional logistic regression was significant, genetic and/or shared familial environmental factors are likely to play a role in the association [29, 31]. In contrast, if the OR in conditional logistic regression remains similar to that from the GEE, and the difference in ORs was not significant, then the confounding by genetic or shared familial environmental factors in the observed association is small or null [24, 30, 32].

Age, sex, education, BMI, smoking, alcohol consumption and marital status were considered as potential confounders. Missing values on education (*n* = 1222), smoking (*n* = 1182), alcohol consumption (*n* = 1253), BMI (*n* = 1801) and marital status (*n* = 757) were imputed using Rubin’s rule for pooling estimates to obtain valid statistical inferences [30]. All statistical analyses were performed using SAS statistical software version 9.4 (SAS Institute, Cary, NC, USA) and IBM SPSS Statistics 20.0 (IBM, New York, NY, USA).

## Results

### Characteristics of the study population

The 33,086 twin individuals included 14,969 (45.2%) men and 18,117 (54.8%) women (χ^2^ = 86.45, *p* < 0.001). Among all participants, 1248 (3.8%) had midlife type 2 diabetes and 3121 (9.4%) had CBD in late life. Compared with type 2 diabetes-free individuals, those with midlife type 2 diabetes were more likely to be younger, male, smokers and alcohol drinkers, and have heart disease, hypertension, and higher BMI (Table 1). Among individuals with midlife type 2 diabetes, those with CBD were older than those without CBD, and there were no significant differences between the two groups in terms of type 2 diabetes treatment (Table 2).

**Table 1:** Characteristics of the study participants by midlife type 2 diabetes mellitus (onset age 40–59 years) (*n* = 33,086)

Characteristics	T2DM-free *n* = 31 838	Midlife T2DM *n* = 1248	*p* value
Age (years), mean (SD)	60.8 (10.3)	58.5 (8.5)	<0.001
Male sex, *n* (%)	14,244 (44.7)	725 (58.1)	<0.001
Education, *n* (%)			0.934
<8 years	11,619 (36.5)	454 (36.4)	
≥8 years	20,219 (63.5)	794 (63.6)	
Marital status, n (%)			0.175
Married/cohabiting	22,933 (72.0)	877 (70.3)	
Single	8905 (28.0)	371 (29.7)	
Zygosity, *n* (%)			0.086
Monozygotic	6323 (19.8)	230 (18.4)	
Dizygotic	21,257 (66.8)	826 (66.2)	
Undetermined	4258 (13.4)	192 (15.4)	
BMI, mean (SD)	24.7 (3.3)	27.6 (4.2)	<0.001
BMI, *n* (%)			<0.001
<20 (underweight)	1847 (5.8)	19 (1.5)	
20–24.9 (normal weight)	16,313 (51.2)	306 (24.5)	
25.0–29.9 (overweight)	11,685 (36.7)	612 (49.0)	
≥30 (obese)	1993 (6.3)	311 (24.9)	
Smoking status, *n* (%)			<0.001
Never smoked	15,670 (49.2)	532 (42.6)	
Former smoker	9031 (28.4)	388 (31.1)	
Current smoker	7137 (22.4)	328 (26.3)	
Alcohol consumption, *n* (%)			<0.001
Never consumed alcohol	29,649 (93.1)	1091 (87.4)	
Past/current consumer of alcohol	2189 (6.9)	157 (12.6)	
Heart disease, *n* (%)	8468 (26.6)	517 (41.4)	<0.001
Hypertension, *n* (%)	8454 (26.6)	693 (55.5)	<0.001

T2DM, type 2 diabetes mellitus

**Table 2:** Clinical features of participants with midlife type 2 diabetes mellitus by CBD (*n* = 1248)

Clinical features	No CBD *n*=1099	CBD *n*=149	*p* value
Age, mean (SD)	57.6 (8.2)	65.1 (7.3)	<0.001
Male sex, *n* (%)	644 (58.6)	81 (54.4)	0.325
T2DM onset age, mean (SD)	52.8 (5.2)	52.4 (4.7)	0.361
T2DM duration, median (IQR)	17.8 (9.2)	18.3 (8.3)	0.534
T2DM treatment, *n* (%)^a^			0.268
Diet	106 (13.0)	10 (7.1)	
Oral glucose-lowering drugs	117 (14.3)	22 (15.6)	
Insulin	36 (4.4)	7 (5.0)	
Combined oral drugs and insulin	558 (68.3)	102 (72.3)	

^a^290 (23.2%) participants had missing values for T2DM treatment

IQR, interquartile range; T2DM, type 2 diabetes mellitus

#### Association between midlife type 2 diabetes and late-life CBD in unmatched case−control analysis

In multi-adjusted GEE models, those with midlife type 2 diabetes had significantly higher ORs of cerebral infarction (OR 1.29 [95% CI 1.03, 1.61]) and occlusion of cerebral arteries (OR 2.03 [95% CI 1.20, 3.44]) in late life than type 2 diabetes-free participants. The associations of midlife type 2 diabetes with subarachnoid haemorrhage (OR 0.52 [95% CI 0.12, 2.21]) and intracerebral haemorrhage (OR 0.78 [95% CI 0.45, 1.36]) were not significant (Table 3).

**Table 3:** ORs and 95% CIs of midlife T2DM in relation to different types of cerebrovascular disease in late life from GEE (T2DM-free as the reference)

CBD	No. of cases	OR (95% CI)^a^	OR (95% CI)^b^	OR (95% CI)^c^
All types of CBD	3121	1.81 (1.50, 2.19)	1.72 (1.42, 2.08)	1.26 (1.04, 1.53)
Cerebral infarction	2190	1.91 (1.54, 2.38)	1.78 (1.43, 2.22)	1.29 (1.03, 1.61)
Occlusion of cerebral arteries	258	2.73 (1.64, 4.55)	2.74 (1.54, 4.60)	2.03 (1.20, 3.44)
Haemorrhagic CBD	540	0.97 (0.59, 1.61)	0.96 (0.58, 1.60)	0.74 (0.44, 1.23)
Subarachnoid haemorrhage	92	0.68 (0.17, 2.78)	0.74 (0.18, 3.07)	0.52 (0.12, 2.21)
Intracerebral haemorrhage	448	1.04 (0.61, 1.79)	1.01 (0.59, 1.73)	0.78 (0.45, 1.36)
Unspecified CBD	133	2.18 (1.04, 4.53)	2.26 (1.11, 4.64)	1.78 (0.87, 3.66)

^a^Adjusted for age, sex and education

^b^Adjusted for age, sex, education, BMI, smoking, alcohol consumption and marital status

^c^Additionally adjusted for hypertension and heart disease

T2DM, type 2 diabetes mellitus

#### Association between midlife type 2 diabetes and late-life cerebral infarction in co-twin matched case−control analysis

In the co-twin matched case−control analysis, the basic- and multi-adjusted ORs for the association between midlife type 2 diabetes and cerebral infarction in late life were 1.60 (95% CI 0.90, 2.84) and 0.96 (95% CI 0.51, 1.80), respectively (Table 4).

**Table 4:** ORs and 95% CIs for the association between midlife T2DM and late-life cerebral infarction in co-twin control analyses using cerebral infarction discordant twin pairs from conditional logistic regression

Co-twin without cerebral infarction	Twin with cerebral infarction	
	T2DM-free	T2DM
T2DM-free	801	32
T2DM	19	7
Basic-adjusted OR (95% CI)^a^	1.60 (0.90, 2.84)	
Multi-adjusted OR (95% CI)^b^	0.96 (0.51, 1.80)	

The 859 cerebral infarction discordant pairs were divided into four groups with respect to exposure (T2DM) status. In 801 twin pairs, both were T2DM-free. In 7 twin pairs, both had T2DM. In 32 twin pairs, the healthy (cerebral infarction-free) co-twin was T2DM-free and the diseased twin had T2DM. In 19 twin pairs, the diseased co-twin was T2DM-free and the healthy twin had T2DM

^a^Adjusted for sex and education

^b^Adjusted for sex, education, BMI, smoking, alcohol consumption, marital status, hypertension and heart disease

T2DM, type 2 diabetes mellitus

The difference in ORs from the GEE model based on unmatched case−control analysis vs co-twin control analysis was not statistically significant (basic-adjusted: OR 1.03 [95% CI 0.67, 1.50; *p* = 0.897]; multi-adjusted: OR 0.94 [95% CI 0.62, 1.39; *p* = 0.780]). Because the estimates from the GEE vs matched co-twin control analysis did not differ significantly, genetic and early-life familial environmental factors appeared not to play a role as confounders in the association between midlife type 2 diabetes and cerebral infarction in late life.

#### Supplementary analysis

Considering possible sex differences in CBD development [2, 33], we performed stratified analyses. The associations between midlife type 2 diabetes and CBD risk and its subtypes did not vary by sex (electronic supplementary material [ESM] Table 1). As midlife type 2 diabetes was associated with mortality (OR 2.70 [95% CI 2.34, 3.11]), we repeated the analysis with an additional adjustment for survival status. The results of this analysis were similar to those of the initial GEE models. Finally, we repeated the analyses excluding data with missing values for covariates, and the results were not significantly altered (ESM Table 2).

## Discussion

In this large-scale, nationwide, population-based study of Swedish twins, we found that midlife type 2 diabetes was significantly associated with increased risk of cerebral infarction and occlusion of cerebral arteries, but not intracerebral haemorrhage or subarachnoid haemorrhage in late life. Genetic and early-life familial environmental factors appeared not to account for the association between midlife type 2 diabetes and late-life cerebral infarction.

Type 2 diabetes may lead to various micro- and macro-vascular changes often culminating in major clinical complications, such as CBD. Epidemiologic studies have shown that type 2 diabetes is associated with a two- to fivefold increased risk of ischaemic CBD, especially cerebral infarction [3, 5, 8, 9, 11–19, 34, 35]. So far, few studies have particularly focused on the association between midlife type 2 diabetes and CBD in late life. In our study, we found that midlife type 2 diabetes conferred a moderate risk for cerebral infarction and occlusion of cerebral arteries in late life.

Several cohort studies have investigated the association of type 2 diabetes with risk of haemorrhagic CBD, and some of them found no such association [3, 5, 7, 14], but others indicated an increased [9] or a decreased [12, 36] risk of haemorrhagic CBD in diabetics. In a longitudinal study examining the relation between type 2 diabetes and haemorrhagic CBD, type 2 diabetes was associated with a decreased subarachnoid haemorrhage risk and an increased intracerebral haemorrhage risk [12]. However, in another two population-based cohort studies, type 2 diabetes was not associated with intracerebral haemorrhage or subarachnoid haemorrhage [3, 18]. The only study addressing the association of midlife type 2 diabetes and haemorrhagic CBD (including intracerebral and subarachnoid haemorrhage) also showed negative results in women [5]. Consistent with these studies, we found that midlife type 2 diabetes was not associated with intracerebral and subarachnoid haemorrhage in late life.

The mechanisms underlying the association of type 2 diabetes with cerebral infarction and occlusion of cerebral arteries are complex and not completely understood [37]. Individuals with diabetes develop dyslipidaemia characterised by small dense low-density lipoproteins, reduced high-density lipoproteins and increased triacylglycerol levels, as well as accelerated atherogenesis [38, 39]. Metabolic disturbances, such as insulin resistance, compensatory hyperinsulinaemia, inflammation, hyperlipidaemia and hyperglycaemia may also contribute to cerebrovascular events in people with type 2 diabetes [37]. The negative association between type 2 diabetes and haemorrhagic CBD may reflect endothelial proliferation and thickening of the basement membrane in cerebral vessels induced by type 2 diabetes that lead to increased risk of infarction but not rupture [40].

Although evidence has shown that adverse socioeconomic circumstances in early life are associated with type 2 diabetes as well as CBD [41–43], the potential contribution of genetic susceptibility to the type 2 diabetes–CBD association is still unknown. In the present study, the lack of significant difference in ORs from the GEE and co-twin control analysis indicates that genetic and unmeasured familial environmental factors (such as maternal nutrition status and childhood socioeconomic situation) appeared not to account for the association between type 2 diabetes and cerebral infarction. However, because of the limited number of cerebral infarction discordant twin pairs in the co-twin analysis, the role of genetic and early-life familial environmental factors in the type 2 diabetes–CBD association needs further investigation.

There are several strengths and limitations in the current study. First, the large nationwide population-based twin cohort provided us with a unique opportunity to examine further the effect of midlife type 2 diabetes on late-life CBD risk, controlling for some unmeasured confounders such as genetic and early-life familial factors. Second, by including only midlife type 2 diabetes and late-life CBD, we could make the temporality clear and thus minimise the possibility of reverse causation. Nonetheless, the limitations in our study need to be pointed out. First, despite the large cohort, the sample size in co-twin matched case−control analysis was limited. Although the association between type 2 diabetes and cerebral infarction in unmatched case−control analysis was attenuated in co-twin matched analysis, the difference was not statistically significant. Thus, caution is needed with regard to the role of genetic and early-life familial environmental factors in the type 2 diabetes–cerebral infarction association. Second, blood glucose levels were not available in the STR or SALT. Consequently, given the higher prevalence of undiagnosed type 2 diabetes in elderly people [44], individuals with undiagnosed type 2 diabetes might have been misclassified as type 2 diabetes-free, which could have led to an underestimation of the association. In addition, participants with midlife type 2 diabetes were about 2 years younger than type 2 diabetes-free individuals. The possible explanations could be that participants with midlife type 2 diabetes could develop CBD before age 60 years and were excluded from the study population, and that patients with midlife diabetes might die before 60. Thus, this might have also led to underestimation for the given association. Third, as both monozygotic twins (who share 100% of their genetic background) and dizygotic twins (who share only 50% of their genetic background) were included in co-twin matched case−control analyses, we could not completely control for the genetic background. Finally, although some lifestyle-related factors (such as smoking and alcohol consumption) and diseases (such as hypertension and heart disease) in midlife were taken into account in the analyses, dietary intake, physical activity and hyperlipidaemia could not be controlled for due to the unavailability of the data among all participants.

In conclusion, our study provides evidence that midlife type 2 diabetes is associated with the risk of cerebral infarction and occlusion of cerebral arteries, but not intracerebral haemorrhage or subarachnoid haemorrhage in late life. Our findings highlight the need to control midlife type 2 diabetes to help prevent cerebral infarction and occlusion of cerebral arteries in late life. Genetic and early-life familial environmental factors appear not to contribute to midlife type 2 diabetes–cerebral infarction association. However, due to the limited sample size in co-twin control analysis, large longitudinal twin studies are warranted for further clarification.

## Electronic supplementary material

ESM Tables (PDF 45 kb)

## Data Availability

Raw data are available by request from qualified investigators applying to the Swedish Twin Registry.

## Abbreviations

CBD: Cerebrovascular disease
GEE: Generalised estimating equation
NPR: National Patient Registry
SALT: Screening Across the Lifespan Twin study
STR: Swedish Twin Registry

## References

[1] World Health Organization (2018) The top 10 causes of death. Available from www.who.int/mediacentre/factsheets/fs310/en/. Accessed 20 Nov 2018

[2] Ji L (2014). Sex disparity in the risk of diabetes-associated stroke. Lancet.

[3] Cui R, Iso H, Yamagishi K (2011). Diabetes mellitus and risk of stroke and its subtypes among Japanese: the Japan public health center study. Stroke.

[4] Myint PK, Sinha S, Luben RN, Bingham SA, Wareham NJ, Khaw KT (2008). Risk factors for first-ever stroke in the EPIC-Norfolk prospective population-based study. Eur J Cardiovasc Prev Rehabil.

[5] Janghorbani M, Hu FB, Willett WC (2007). Prospective study of type 1 and type 2 diabetes and risk of stroke subtypes: the Nurses’ Health Study. Diabetes Care.

[6] Almdal T, Scharling H, Jensen JS, Vestergaard H (2004). The independent effect of type 2 diabetes mellitus on ischemic heart disease, stroke, and death: a population-based study of 13,000 men and women with 20 years of follow-up. Arch Intern Med.

[7] Abbott RD, Donahue RP, MacMahon SW, Reed DM, Yano K (1987). Diabetes and the risk of stroke. The Honolulu Heart Program. JAMA.

[8] Iso H, Imano H, Kitamura A (2004). Type 2 diabetes and risk of non-embolic ischaemic stroke in Japanese men and women. Diabetologia.

[9] Sarwar N, Gao P, Emerging Risk Factors Collaboration (2010). Diabetes mellitus, fasting blood glucose concentration, and risk of vascular disease: a collaborative meta-analysis of 102 prospective studies. Lancet.

[10] Luitse MJ, Biessels GJ, Rutten GE, Kappelle LJ (2012). Diabetes, hyperglycaemia, and acute ischaemic stroke. Lancet Neurol.

[11] Larsson SC, Scott RA, Traylor M (2017). Type 2 diabetes, glucose, insulin, BMI, and ischemic stroke subtypes: Mendelian randomization study. Neurology.

[12] Shah AD, Langenberg C, Rapsomaniki E (2015). Type 2 diabetes and incidence of cardiovascular diseases: a cohort study in 1.9 million people. Lancet Diabetes Endocrinol.

[13] Khoury JC, Kleindorfer D, Alwell K (2013). Diabetes mellitus: a risk factor for ischemic stroke in a large biracial population. Stroke.

[14] Murakami Y, Huxley RR, Lam TH (2012). Diabetes, body mass index and the excess risk of coronary heart disease, ischemic and hemorrhagic stroke in the Asia Pacific Cohort Studies Collaboration. Prev Med.

[15] Banerjee C, Moon YP, Paik MC (2012). Duration of diabetes and risk of ischemic stroke: the Northern Manhattan Study. Stroke.

[16] O’Donnell MJ, Xavier D, Liu L (2010). Risk factors for ischaemic and intracerebral haemorrhagic stroke in 22 countries (the INTERSTROKE study): a case-control study. Lancet.

[17] Doi Y, Ninomiya T, Hata J (2010). Impact of glucose tolerance status on development of ischemic stroke and coronary heart disease in a general Japanese population: the Hisayama study. Stroke.

[18] Tanizaki Y, Kiyohara Y, Kato I (2000). Incidence and risk factors for subtypes of cerebral infarction in a general population: the Hisayama study. Stroke.

[19] Folsom AR, Rasmussen ML, Chambless LE (1999). Prospective associations of fasting insulin, body fat distribution, and diabetes with risk of ischemic stroke. The Atherosclerosis Risk in Communities (ARIC) Study Investigators. Diabetes Care.

[20] Knekt P, Reunanen A, Aho K (1991). Risk factors for subarachnoid hemorrhage in a longitudinal population study. J Clin Epidemiol.

[21] Kyvik KO, Green A, Beck-Nielsen H (1995). Concordance rates of insulin dependent diabetes mellitus: a population based study of young Danish twins. BMJ.

[22] Bak S, Gaist D, Sindrup SH, Skytthe A, Christensen K (2002). Genetic liability in stroke: a long-term follow-up study of Danish twins. Stroke.

[23] Brass LM, Isaacsohn JL, Merikangas KR, Robinette CD (1992). A study of twins and stroke. Stroke.

[24] Xu W, Qiu C, Gatz M, Pedersen NL, Johansson B, Fratiglioni L (2009). Mid- and late-life diabetes in relation to the risk of dementia: a population-based twin study. Diabetes.

[25] Lundqvist E, Kaprio J, Verkasalo PK (2007). Co-twin control and cohort analyses of body mass index and height in relation to breast, prostate, ovarian, corpus uteri, colon and rectal cancer among Swedish and Finnish twins. Int J Cancer.

[26] Lichtenstein P, De Faire U, Floderus B, Svartengren M, Svedberg P, Pedersen NL (2002). The Swedish Twin Registry: a unique resource for clinical, epidemiological and genetic studies. J Intern Med.

[27] Chinese Medical Association (2017) Classification of cerebrovascular disease (2015). Chin J Neurol 168–171. 10.3760/cma.j.issn.1006-7876.2017.03.003 [article in Chinese]

[28] Gatz M, Svedberg P, Pedersen NL, Mortimer JA, Berg S, Johansson B (2001). Education and the risk of Alzheimer’s disease: findings from the study of dementia in Swedish twins. J Gerontol B Psychol Sci Soc Sci.

[29] Kato K, Sullivan PF, Evengard B, Pedersen NL (2006). Premorbid predictors of chronic fatigue. Arch Gen Psychiatry.

[30] Xu WL, Atti AR, Gatz M, Pedersen NL, Johansson B, Fratiglioni L (2011). Midlife overweight and obesity increase late-life dementia risk: a population-based twin study. Neurology.

[31] Bao C, Pedersen NL, Yang R (2018). Diabetes in midlife and risk of cancer in late life: a nationwide Swedish twin study. Int J Cancer.

[32] Bao C, Yang R, Pedersen NL (2019). Overweight in midlife and risk of cancer in late life: a nationwide Swedish twin study. Int J Cancer.

[33] Peters SA, Huxley RR, Woodward M (2014). Diabetes as a risk factor for stroke in women compared with men: a systematic review and meta-analysis of 64 cohorts, including 775,385 individuals and 12,539 strokes. Lancet.

[34] Chen R, Ovbiagele B, Feng W (2016). Diabetes and stroke: epidemiology, pathophysiology, pharmaceuticals and outcomes. Am J Med Sci.

[35] Kissela BM, Khoury J, Kleindorfer D (2005). Epidemiology of ischemic stroke in patients with diabetes: the Greater Cincinnati/Northern Kentucky Stroke Study. Diabetes Care.

[36] Karapanayiotides T, Piechowski-Jozwiak B, van Melle G, Bogousslavsky J, Devuyst G (2004). Stroke patterns, etiology, and prognosis in patients with diabetes mellitus. Neurology.

[37] Air EL, Kissela BM (2007). Diabetes, the metabolic syndrome, and ischemic stroke: epidemiology and possible mechanisms. Diabetes Care.

[38] Lee M, Saver JL, Hong KS, Song S, Chang KH, Ovbiagele B (2012). Effect of pre-diabetes on future risk of stroke: meta-analysis. BMJ.

[39] Howard BV, Robbins DC, Sievers ML (2000). LDL cholesterol as a strong predictor of coronary heart disease in diabetic individuals with insulin resistance and low LDL: the Strong Heart Study. Arterioscler Thromb Vasc Biol.

[40] Alex M, Baron EK, Goldenberg S, Blumenthal HT (1962). An autopsy study of cerebrovascular accident in diabetes mellitus. Circulation.

[41] Jornayvaz FR, Vollenweider P, Bochud M, Mooser V, Waeber G, Marques-Vidal P (2016). Low birth weight leads to obesity, diabetes and increased leptin levels in adults: the CoLaus study. Cardiovasc Diabetol.

[42] Lawlor DA, Ronalds G, Clark H, Smith GD, Leon DA (2005). Birth weight is inversely associated with incident coronary heart disease and stroke among individuals born in the 1950s: findings from the Aberdeen Children of the 1950s prospective cohort study. Circulation.

[43] Hart CL, Hole DJ, Smith GD (2000). Influence of socioeconomic circumstances in early and later life on stroke risk among men in a Scottish cohort study. Stroke.

[44] Gregg EW, Cadwell BL, Cheng YJ (2004). Trends in the prevalence and ratio of diagnosed to undiagnosed diabetes according to obesity levels in the U.S.. Diabetes Care.

