# Supplementary material for: Type 2 diabetes in midlife and risk of cerebrovascular disease in late life: a prospective nested case−control study in a nationwide Swedish twin cohort
Source: Diabetologia. 2019 Jun 5;62(8):1403–11. doi: 10.1007/s00125-019-4892-3 (PMC6647245; doi:10.1007/s00125-019-4892-3)
Supplement: Supplementary file 1 — (PDF 45 kb) [file 125_2019_4892_MOESM1_ESM.pdf]

Electronic supplementary material (ESM)

ESM Table 1. Odds ratios (ORs) and 95% confidence intervals (CIs) of midlife type 2 diabetes mellitus (T2DM) related to different forms of late-life cerebrovascular disease (T2DM-free as the reference) from Generalized Estimating Equation models among men and women

Cerebrovascular Disease (CBD)	Male			Female		
	Cases	OR (95% CI)[a]	OR (95% CI)[b]	Cases	OR (95% CI)[a]	OR (95% CI)[b]
All types CBD	1563	1.55 (1.21, 1.99)	1.05 (0.81,1.36)	1558	2.23 (1.67, 2.98)	1.60 (1.20, 2.15)
Cerebral infarction	1079	1.66 (1.24, 2.22)	1.09 (1.03, 1.48)	1111	2.29 (1.64, 3.19)	1.59 (1.13, 2.22)
Occlusion of cerebral arteries	141	2.15 (1.08, 4.30)	1.53 (1.01, 3.13)	117	3.76 (1.77, 7.97)	3.02 (1.40, 6.51)
Hemorrhagic CBD	279	0.95 (0.50, 1.79)	0.66 (0.34, 1.27)	261	1.00 (0.44, 2.28)	0.83 (0.36, 1.91)
Subarachnoid hemorrhage	41	0.59 (0.08, 4.38)	0.42 (0.06, 3.13)	51	0.79 (0.11, 5.76)	0.66 (0.08, 5.10)
Intracerebral hemorrhage	238	1.02 (0.52, 2.00)	0.71 (0.36, 1.41)	210	1.06 (0.43, 2.61)	0.88 (0.36, 2.17)
Unspecified CBD	64	1.45 (0.46, 4.65)	1.13 (0.35, 3.69)	69	3.31 (1.31, 8.34)	2.77 (1.16, 6.66)

[a] Adjusted for age and education.
[b] Adjusted for age, education, body mass index, smoking, alcohol consumption, marital status, hypertension and heart disease.

ESM Table 2. Odds ratios (ORs) and 95% confidence intervals (CIs) of midlife type 2 diabetes mellitus (T2DM) related to different forms of late-life cerebrovascular disease (T2DM-free as the reference) from Generalized Estimating Equation models using data available (excluding data with missing values for covariate)

Cerebrovascular Disease (CBD)	No. of Cases	OR (95% CI)[a]
All types CBD	2690	1.30 (1.06, 1.59)
Cerebral infarction	1903	1.34 (1.06, 1.69)
Occlusion of cerebral arteries	198	1.89 (1.07, 3.35)
Hemorrhagic CBD	481	0.81 (0.48, 1.36)
Subarachnoid hemorrhage	87	0.58 (0.14, 2.47)
Intracerebral hemorrhage	394	0.86 (0.50, 1.49)
Unspecified CBD	108	2.15 (1.04, 4.45)

[a] Adjusted for age, sex, education, body mass index, smoking, alcohol consumption, marital status, hypertension and heart disease.
